# Schema therapy for borderline personality disorder: A qualitative study of patients’ perceptions

**DOI:** 10.1371/journal.pone.0206039

**Published:** 2018-11-21

**Authors:** Yeow May Tan, Christopher W. Lee, Lynn E. Averbeck, Odette Brand-de Wilde, Joan Farrell, Eva Fassbinder, Gitta A. Jacob, Desiree Martius, Sophie Wastiaux, Gerhard Zarbock, Arnoud Arntz

**Affiliations:** 1 Department of Psychology and Exercise Science, Murdoch University, Perth, Australia; 2 Department of Health and Medical Sciences, University of Western Australia, Perth, Australia; 3 Institute of Psychology, University of Hamburg, Hamburg, Germany; 4 De Viersprong, Netherlands Institute of Personality Disorders, Halsteren, The Netherlands; 5 Department of Psychology, Indiana University-Purdue University Indianapolis, Indianapolis, United States of America; 6 Department of Psychiatry and Psychotherapy, University of Lübeck, Lübeck, Germany; 7 Department of Clinical Psychology and Psychotherapy, University of Freiburg, Freiburg, Germany; 8 Department of Clinical Psychology, University of Amsterdam, Amsterdam, The Netherlands; 9 IVAH - Institut für Verhaltenstherapie-Ausbildung, Hamburg, Germany; Universita degli Studi di Padova Dipartimento di Ingegneria Industriale, ITALY

## Abstract

Schema therapy (ST) has been found to be effective in the treatment of borderline personality disorder (BPD). However very little is known about how the therapy is experienced by individuals with BPD including which specific elements of ST are helpful or unhelpful from their perspectives. The aim of this study is to explore BPD patients’ experiences of receiving ST, in intensive group or combined group-individual format. Qualitative data were collected through semi-structured interviews with 36 individuals with a primary diagnosis of BPD (78% females) who received ST for at least 12 months. Participants were recruited as part of an international, multicenter randomized controlled trial (RCT). Interview data (11 Australian, 12 Dutch, 13 German) were analyzed following the procedures of qualitative content analysis. Patients’ perceptions of the benefits gained in ST included improved self-understanding, and better awareness and management of their own emotional processes. While some aspects of ST, such as experiential techniques were perceived as emotionally confronting, patient narratives informed that this was necessary. Some recommendations for improved implementation of ST include the necessary adjunct of individual sessions to group ST and early discussion of therapy termination. Implications of the findings are also discussed, in particular the avenues for assessing the suitability of patients for group ST; management of group conflict and the optimal format for delivering treatment in the intensive group versus combined group-individual formats.

## Introduction

Borderline personality disorder (BPD) is a pervasive, debilitating psychological condition found in 28.5% of clinical populations and between 0.8% and 1.6% among community samples [1, 2]. BPD is often regarded as the most lethal form of mental illness and the most severe personality disorder [3], where completed suicides are estimated between 8.5% and 10% [4, 5]. Such patients are considered high treatment utilizers as they consume extensive treatment resources, and a large proportion return to therapy after termination of the initial treatment [6]. Over recent decades, specialist psychological treatments including Dialectical Behavioural Therapy (DBT), Mentalization Based Therapy (MBT), Transference Focused Psychotherapy (TFP) and Schema Therapy (ST) have shown to be of benefit in treating BPD [7].

ST was developed as a treatment for personality disorders and other chronic symptom disorders. It is an integrative form of psychotherapy that incorporates concepts and approaches from Cognitive Behaviour Therapy (CBT), attachment theory, gestalt therapy and psychodynamic perspectives. It relies on two conceptual models in the formulation of patients’ issues and to understand the change process. The first is Early Maladaptive Schemas (EMS), defined as pervasive and self-defeating, dysfunctional patterns of thoughts, cognitions, behaviours and affects that typically develop during childhood but become elaborated on throughout the person’s life [8]. These EMS develop when there is a mismatch between a child’s basic needs and their environment. Eighteen EMS have been identified but it is also possible to categorize these into a hierarchy that consists of domains such as the need for connection, autonomy and reasonable limits [9]. Patients with BPD have been found with elevated scores on most schemas compared to other population groups, particularly in need for connection domain [10].

Second, the schema mode model was developed on the basis that individuals with BPD often experienced rapid emotional changes with simultaneous activations of several schemas [8]. The mode model describes current states rather than traits; at any one point in time the mode refers to the interaction between an individual’s schemas and their coping style. Researchers have identified four problematic modes common in BPD patients [11]. For example the mode that represents their core distress has been labelled vulnerable (abandoned-abused) child mode. In this mode the patient’s feelings are in the rawest state, where they feel intensely worthless, unlovable, helpless, incompetent or abandoned. They frequently feel overwhelmed and look to others for solutions. According to the theory given the adversity of such a state, the patient will typically move from this to an alternative state. In BPD this might be an angry or an impulsive child mode. In angry mode the patient demands that others fix the situation or in impulsive child mode the patient tries to change the underlying pain through self-gratification impulses with little or no regard to consequences. An alternative way of dealing with the distress associated with the vulnerable child mode is to use dissociation or other mechanisms of detachment; these symptoms are part of a state labelled the detached mode. Activation of the vulnerable child mode can also lead to an activation of the punitive parent mode, through which the patient experiences harsh, punitive thoughts (or a voice) about the self including feelings of shame, guilt and self-loathe [11]. This mode is thought to result from the internalization of their caregivers’ punitive messages. Typically, BPD patients subsequently shift to the detached protector mode to split off from the emotional pain associated with the punitive parent mode [11]. Many patients remain largely in the detached protector mode to prevent activation of the other modes in the absence of more functional ways to deal with these modes (i.e. their healthy adult mode remains underdeveloped).

Therapy includes psychoeducation of BPD symptoms as they relate to modes, including how each mode plays out in the patient’s life. The next phase is to provide experiences in the therapy of soothing the patient’s vulnerable child mode, identifying the needs of angry impulsive child mode while setting limits and helping to fight the destructive messages of the punitive parent mode. The goal of therapy is to develop the patient’s ability to do this for themselves by providing new experiences in session. The use of experiential techniques have been found to distinguish ST from CBT and psychodynamic therapies [12]. Experiential techniques including imagery rescripting and chair work dialogues have been linked to improving emotional dysregulation in personality disorders [13]. Imagery involves recalling a particular situation and the events that occurred, together with one’s thoughts and feelings. Imagery rescripting is a method that subsequently assists in re-creating and altering parts of that situation in order to change one’s experience and meaning of that past situation [14]. On the other hand, the use of chair work allows for dialogues and interactions with significant persons in the re-enactment of a past situation, and is particularly useful in increasing the metacognitive capacity of individuals when working with their respective modes [15]. Both experiential techniques serve as agents in facilitating one’s access to emotions and unmet core needs [13].

ST has shown particular promise as a highly effective and acceptable treatment. To date nine published studies have demonstrated the effectiveness of individual and/or group ST (GST) in ameliorating borderline symptomatology and improving quality of life. Three studies were randomized controlled trials (RCT); one study contrasted ST with TFP [16], another with treatment-as-usual [17], and the other assessed whether telephone availability of therapists outside office hours added to ST effectiveness in regular healthcare settings [18]. Six other studies were uncontrolled trials, including a case series study [19], an open trial piloting a combined group and individual format [20], a feasibility study of GST for outpatients with high severity BPD [21] and three open trials on inpatient ST for BPD [22]. Given some indication that GST might be especially beneficial for BPD patients [17, 20], the present study aimed to further explore specific aspects of ST that comprise this benefit, particularly beyond existing quantifiable measures.

There has been increasing interest in consumers’ perspectives regarding their experience of treatment. A meta-analysis of 14 qualitative studies revealed BPD patients’ experience of positive changes in their respective treatments including improved confidence, better relationships and ability to regulate emotions supported by a sense of safety and containment, feeling respected, and feeling included in treatment decisions [23]. The treatments specified in the studies were delivered in an individual or group format, or a combination of both; these included DBT, MBT, art therapy, peer support group, and standard community mental health services [22]. However there has only been one qualitative paper on the experiences of participants receiving ST for treatment of a personality disorder [24]. In that study participants described helpful aspects of the treatment as the therapy relationship, specific schema techniques, and self-understanding facilitated by the schema model. Unhelpful aspects reported included perceived lack of preparation for strong negative emotions generated in the therapy and insufficient number of sessions. Whilst these patients all met criteria for a personality disorder, none of them had BPD [24].

In summary to date, there is an absence of qualitative information on how ST and GST is experienced by BPD patients. Such information is important as qualitative methods enable an understanding of how the therapy is received by patients which is a perspective not often available by simply measuring symptoms or quality of life ratings. This study explored such experiences, in particular:

1. Aspects of individual and group ST that were helpful (unhelpful) in facilitating (hindering) recovery from BPD.
2. Group dynamics and processes among BPD patients within GST.
3. Patients’ opinions about the ST structure and respective formats (i.e. group- or combined group-individual ST) to inform more optimal delivery of ST for BPD.

## Method

### Participants

Participants in this study were recruited from the ST treatment condition of an international multicentre RCT conducted in 14 sites across five countries [25]. Inclusion criteria included participants aged between 18 and 65 years and meeting criteria for a primary diagnosis of BPD. Participants were excluded if they met criteria for lifetime psychotic disorder, ADHD, Bipolar Disorder Type I, Dissociative Identity Disorder, Narcissistic or Antisocial PDs. More information on how participants’ diagnoses were determined can be found in the design article [25]. Diagnoses were made based on the Structured Clinical Interviews for DSM-IV Axis I and II diagnoses (SCID-I; SCID-II).

Participants receiving this therapy were sought from eight sites across three countries; Australia, Germany and the Netherlands. Every participant at the chosen sites was approached to participate. Of the 45 approached, 39 agreed to take part. One did not attend the scheduled interview and one attended but did not engage adequately and the session was terminated. One interview was not correctly recorded and was excluded. Data from the remaining 36 participants (eight males, 28 females) were included in the analyses. During the time of interview, these participants had all passed the halfway point of the ST treatment protocol and had therefore received ST for at least 12 months and up to 24 months. They comprised 11 Australians, 12 Dutch, and 13 Germans. All but two participants met the diagnostic criteria for at least one other disorder. Most common axis one comorbidities were affective disorders (92%), anxiety disorders (67%), substance disorders (39%), eating disorders (31%), and other axis one disorders (8%). Over half of the participants (56%) also met criteria for another personality disorder.

Fifteen of the 36 participants received a combination of weekly group and individual ST for the first 12 months, which decreased to fortnightly sessions in the next six months and subsequently monthly sessions in the remaining six months. The total number of sessions scheduled for these participants were 136. Twenty-one participants received twice-weekly group ST with an optional bank of 12 individual ST sessions in the first 12 months, which decreased to weekly group ST in the next six months followed by fortnightly group sessions for three months and subsequently monthly group ST in the last three months. Participants also had an optional bank of six individual ST sessions in the last year, which they could request from the group therapist. The participants in this condition used all their optional individual sessions in the first 12 months, some did not use up their allocated sessions in the second year and the range of scheduled sessions was between 134 and 140 over the two years.

### Procedure

In-depth semi-structured interviews were conducted and a topic list was constructed after a pilot interview with a service user and various discussions with the research team, which comprise researchers with academic backgrounds and clinical expertise. None of the interviewers were involved in the treatment delivery process. Interviews started with open questions on patients’ general experiences, reflections and opinions about their therapy. For each topic, examples of open-ended questions were used as shown in Table 1; however there was no obligation for the interviewer to use them, especially when topics were addressed spontaneously by the patient. Similarly if patients indicated nil differences in their experience between group and individual therapy, they would not be prompted to further elaborate on these (e.g. extent their needs are met in either). A flexible interview style was adopted and the topic list served to guide rather than dictate the interview. Other areas that patients perceived as significant, but not covered in the topic list were also explored.

**Table 1:** Examples of interview questions in topic list.

**Overall experiences**
How was your experience of schema therapy?
How were other therapies you’ve done different from schema therapy?
**Group experiences**
How was your experience of group schema therapy?
How did you find talking and sharing about your feelings and emotions to other group members?
**Group vs Individual therapy experiences**
To what extent did you respond differently in group and in individual therapy?
To what extent did you feel your needs were met in group and individual therapy?
**Therapeutic tasks**
How were discussion topics decided?

The interviews lasted between 60 and 180 minutes. All but one interview were audio-recorded with a digital recorder and transcribed verbatim. The unrecorded interview was conducted on prison grounds and interview content was recorded using shorthand. To preserve the integrity and authenticity of the data informant feedback was sent to each patient to comment and correct if necessary. These comments and corrections were added to the transcripts and incorporated in the analyses.

All participants provided written informed consent following verbal and written explanation of the study information. The patient who was incarcerated agreed to participate via reply paid post of the forms sent to the prison. It has been outlined in the information letters that participants can decide at any time to withdraw their consent to participate in the study without any impact on the treatment received. The research protocol has been approved by the Medical Ethics Committee of Maastricht University for the Dutch sites; the Murdoch University Human Research Ethics Committee for the Australian sites; and the Ethics Committee of the Albert-Ludwigs-University Freiburg, the Ethics Committee of the University of Lübeck and the Ethics Committee of the Psychotherapist Association Hamburg for the German sites.

### Data analysis

Interview data were analyzed following the systematic, analytic procedures of qualitative content analysis. The approach is used to “systematically describe the meaning” of materials [26] based on our research questions formulated beforehand, as represented in the Topic List shown in Table 1, and other specific themes that emerge. These topic questions were to guide the interview and systematically capture the patients’ experiences in their therapy. Qualitative content analysis often utilizes a combination of inductive and deductive approaches, and a data reduction process where the analysis is limited to selected aspects relevant to the research questions [26]. Data analysis began with repeated readings of the interview transcripts from an unbiased perspective to gain familiarity and generate an overall impression of the data. Relevant passages or texts that came across as meaningful were then extracted and organized into categories and subcategories, not limited to issues related to the apriori-formulated topics.

The first author (YMT) and another author (DM) developed the preliminary coding frame together. The process allowed for clarification regarding naming of each category/theme and the appropriate placement of each interview passage. Two other authors (CWL and AA) also provided feedback and helped refine the labelling of the main themes. Subsequently, the core analyses of the transcripts were conducted with the use of MAXQDA [27]. Two authors (SW and YMT) each coded a total of 21 transcripts (i.e. seven Dutch, seven German, seven Australian) not used in the initial development of themes. Datasets on MAXQDA were exported and merged to calculate the inter-coder agreement, or Cohen’s Kappa [28].

To calculate Cohen’s Kappa for each topic/category, a contingency table was created utilizing a 95 percent confidence interval. MAXQDA generated an output table with all segments jointly coded for each topic displaying the segments agreed by both coders, segments coded by first (YMT) but not second rater (SW), and segments coded by second but not the first rater. These were recorded in the contingency table together with the total number of coded segments (i.e. 2037) and Cohen’s Kappa was calculated for each topic within the coding frame. While there exists some variance on what constitutes an appropriate cut-off for Cohen’s Kappa, an estimated value of at least .70 is sufficient for good inter-coder reliability [29]. Disagreements were resolved by further analyzing transcripts with the lowest agreement by coded segments. The process of deriving the final Kappa values took approximately eight hours of discussion via Skype. Results of inter-coder reliability for each topic/category are displayed in Table 2. Once saturation of the themes and satisfactory inter-coder agreement were achieved, YMT coded the remaining transcripts.

**Table 2:** Inter-coder agreement for each topic/category.

Topic/Category	Cohen’s Kappa
Benefits gained and challenges faced in ST	.73
Perceptions of ST as compared to previous therapies	.74
Group experiences and dynamics	.71
Structure and format of therapy	.75
Therapeutic relationships	.80

## Results

Various themes relating to patients’ experiences of ST were discussed and are summarized in Table 3; beginning with the benefits gained and challenges faced, followed by patients’ perceptions of ST compared to past therapy experiences. Patients also reflected their observations and interactions within the group, specific components and structural aspects of group and individual ST, and finally the therapeutic relationships. Examples of quotations selected from particular patients have been included throughout this section.

**Table 3:** Broad perceptions of group and individual components of schema therapy.

Topics/Themes	N (%)
**A. Benefits gained and challenges faced in ST**	
A1. Extent to which ST provided insight	31 (86)
A2. Ability to act differently and cope adaptively	26 (72)
A3. Changes in connection with one’s emotions	18 (50)
A4. Change in confidence levels and assertiveness	17 (47)
A5. Extent to which ST minimized harshness to the self	15 (42)
A6. Necessity of difficulty level in ST	14 (39)
**B. Perceptions of ST as compared to previous therapies**	
B1. Degree of focus on internal processes	16 (44)
B2. Extent to which ST was prescriptive vs. tailored to individual needs	11 (31)
**C. Group experiences and dynamics**	
C1. Sense of connection among group members	33 (92)
C2. Extent to which one felt safe, accepted and able to trust others	28 (78)
C3. Feelings that arose when comparing oneself against others	18 (50)
C4. Gender composition of the group	18 (50)
**D. Structure and format of therapy**	
D1. Extent to which group and individual ST complemented each other	27 (75)
D2. The use of experiential techniques	25 (69)
D3. Duration/length of therapy sessions	22 (61)
D4. The use of schema-mode model	21 (58)
D5. Email access to therapists outside working hours	13 (36)
**E. Therapeutic relationships**	
E1. Extent to which patients feel supported by their therapists	29 (81)

### A. Benefits gained and difficulties faced in ST

While most patients reported therapeutic gains due to ST as described in the themes below, 14 of them (39%) also commented on difficulties.

#### A1. Extent to which ST provided insight

All but five patients (86%) described how ST facilitated understanding of the self and internal processes while making sense of their problems and reactions to certain situations. Prior to ST patients tended to respond in disproportionate or inappropriate ways to certain situations or with maladaptive behaviour patterns including aggression and impulsivity. ST was indicated as helpful in providing explanations on the borderline condition and assisting patients to recognize their triggers. *“With ST we started off by getting an understanding of our condition and that gives you more of a knowledge of why you’re reacting the way you’re reacting or why you’re doing the things you’re doing rather than that’s just because you’ve got depression- and for me*, *that makes a lot more sense… It’s like explaining to a diabetic why their body doesn’t produce the insulin*, *it’s like explaining to us why borderline personality has come about”* (patient 5206). For some (*n* = 9) the improved insight increased their ability to change their behaviours. *“When I understand what is going on inside me*, *how these processes are… I can structure all of that really well for myself and then also deal with it better”* (patient 2411).

#### A2. Ability to act differently and cope adaptively

Twenty-six patients (72%) described greater capacity to cope and apply skills learnt, without turning to less helpful ways of coping. Specifically, patient narratives included improved motivation to gradually achieve one’s goals and reduce self-harm and suicidal behaviours. *“There’re actually a lot of problem behaviours which I had; the self-injuring behaviour*, *drinking… They all somehow more or less disappeared- that is a stupid word*, *because it doesn’t disappear*, *but it just somehow changed*. *I don’t display obvious symptom behaviours anymore and also the emotional instability- this up and down*, *I do not have anymore*. *My mood completely stabilized and I also stopped taking all my medications”* (patient 2018).

#### A3. Changes in connection with one’s emotions

Half of the patients mentioned how ST allowed them to get in touch or reconnect with the feelings or emotions that had previously been blocked off. The increased connection with and/or awareness of one’s emotions was generally described as a shift from intellectualizing to experiencing them. *“I could sit there and tell you about all the times it (*Traumatic incident in patient 5002’s past) *happened but I wouldn’t connect to it emotionally*, *and schema sort of reconnected the emotional side of me*, *I could reconnect with that feeling of being hurt and that it wasn’t right… I was intellectualizing it too much*, *I wanted to understand it and that was frustrating”* (patient 5002).

#### A4. Change in confidence levels and assertiveness

Patients (*n* = 17; 47%) described that ST improved their confidence to speak up for themselves and accomplish what they were unable to do before. Ten of them further described some flexibility and openness in experimenting with asserting one’s needs. *“I remember I had this fight with my partner where I was like*, *‘You’re not going to leave me just because we had a fight’ so I’m going to be assertive and put my thoughts forward without having to do all the passive-aggressive stuff*. *I did it and I was like*, *‘Ok*. *The world didn’t end*. *That’s a first and you didn’t leave me*, *I actually got my point across for once”* (patient 5033). Eleven patients indicated a general increase in confidence levels in facing their difficulties rather than avoiding them. They described reduced feelings of fear toward vulnerable parts of themselves, increased acceptance and willingness to be vulnerable in therapy and participate in experiential techniques. *“I’m always afraid to close my eyes and imagine something- to put myself back into old situations because they make me afraid*. *I do know that the changes are good*, *but first it is still loaded with fear*, *and then now it does not scare me as much”* (patient 2404).

#### A5. Extent to which ST minimized harshness to the self

Fifteen patients (42%) described feeling less harsh toward themselves particularly after gaining an understanding of where it stemmed from. They described regaining some sense of control over the punitive parent mode or the harsh voices they had internalized since childhood *“Back then the punishing voices were a constant part in my head and the memories of the people that are represented by these voices come up*. *That is not the case anymore*. *Today I only see the words*, *the sentences*, *without the memories coming up”* (patient 2404). They also appear more able to separate from demands placed on them without blaming themselves when these demands are not fulfilled. *“The most important thing for me was to be softer to myself… I was very demanding to myself and to others*. *I listened a lot to my ‘punisher’ and he said very ugly things to me*. *I’ve learnt to defend myself- there are still moments that my ‘punisher’ is very active*, *but I have the capacity to shoot him down*. *That feeling of being no good*, *not good enough- that feeling’s become less*!*”* (patient 7006).

#### A6. Necessity of difficulty level in ST

While aspects of ST had been described as difficult, 14 patients (39%) found the process necessary and helpful. An array of descriptors were used to convey the difficulty of ST, which included “overwhelming”, “scary”, “painful”, “emotionally, psychological confronting”, “draining” and one patient described the process as *“sticking their hand in the hot oven”* (patient 5211). Such challenging experiences generally involved the process of revisiting traumatic events that occurred in the past and getting to know their emotions and vulnerable selves. *“I always pushed down a lot of memories because they’re too painful for a child to remember*. *Schema helped to bring them out but I couldn’t have received treatment without acknowledging the existence of the trauma and I had to bring the trauma out on the table- to work on them”* (patient 5224).

### B. Perceptions of ST as compared to previous therapies

All patients compared and contrasted ST to their prior experience of other therapy approaches, and have attributed differences largely to the therapy model; these included the depth afforded by schema concepts (e.g. modes) and its consideration of one’s unique background. Despite ST being perceived as more difficult for five patients, a majority of them (*n* = 23; 64%) preferred ST and 22 of them (61%) found the ST model more effective than other therapies.

#### B1. Degree of focus on internal processes

Sixteen patients (44%) indicated that ST delves deeper into possible reasons for one’s unique ways of thinking, feeling and behaving compared to their past therapies, primarily DBT. *“You just learn in DBT how to survive with skills*, *while in ST*, *you come to think and therefore also could aim at changes*, *and not only to distract*, *to stop injuring myself but also via the thinking-level”* (patient 2219). They generally found the mode model more effective for more extensive problems, and not just specific to those with a borderline diagnosis. ST was described as more confronting and scary, particularly having to revisit one’s past trauma and being vulnerable within the group. *“DBT has nothing on how confronting this can be because it’s working from the outside-in rather than inside-out*. *ST focuses more like on your inside parts*. *With DBT you can calm yourself with like mindfulness or meditation whereas with schema it’s very different because we’re being taught the opposite*, *to focus on why you’re doing what you’re doing… I mean that (*Dialectical Behavioural Therapy) *does help but I’m just saying this* (Schema Therapy) *helped more but it’s a harder road to get to the point where it starts to help”* (patient 5211).

#### B2. Extent to which ST was prescriptive vs. tailored to individual needs

Previous therapies were seen as prescriptive and authoritative in encouraging patients to practise skills learnt in therapy (*n* = 11; 31%), ST on the other hand provided reasoning to the importance of practising these skills, exploring the origins of one’s difficulties and where to go from there. *“ST is a lot more individually tailored*. *You look at where are the main focus points; in what mode are you most of the time*? *Where do we have to work on the most*? *In the others it was always only to look for a skill*! *Those skills do not help me*, *I cannot look for some skills the whole day”* (patient 2456). In this sense, ST is not a ‘one size fits all’ therapy as articulated by one patient but is perceived to consider the unique needs of each patient. *“In ST*, *you have your one-on-one sessions which hashes out why you’re struggling with certain things or why you find things easier than others or why you can’t recognize certain modes*. *It’s not a cookie cutter it can be tailored to each person even if you’re doing the group”* (patient 5211).

### C. Group experience and dynamics among group members

All patients discussed and frequently referred to their experiences as a group member. Apart from some initial discomfort, the process of sharing similar experiences was positively rated. Other negative group interactions included feeling excluded and feeling frustrated compared to others who seemed to be progressing. There were also several patients who felt unsafe to fully express themselves following unpleasant incidents and conflicts that occurred during group.

#### C1. Sense of connection among group members

Thirty-two patients (89%) discussed the sense of connection among group members where more than half (*n* = 24; 67%) believed that being in the company of similar others allowed them to bond and develop an understanding that they were not alone in experiencing such difficulties. One of them described feeling more visible through this process. *“At the beginning I always sat close to the door*, *and then during the therapy I went sitting further and further away* (from the door). *I felt more connected to the group because I felt they saw me*. *You see others with the same problems and I thought*: *Oh*! *Others feel the same*! *I always thought*: *that’s me*, *I’m strange*!*”* (patient 7426). On the other hand, a minority (*n* = 6; 17%) described feeling left out and not understood as they believed they had “nothing in common” with other group members due to being in different stages of life. *“No one else works so it’s like no one can understand that I’ve got other stuff going on outside… sometimes I feel as though I’m outside looking in… I had a lot of stuff going on at work and when people don’t work they will say ‘well just give up your job’ they just don’t understand”* (patient 5221).

#### C2. Extent to which one felt safe, accepted and able to trust others

Feelings of safety within the group and trust among group members were salient for 28 patients (78%). Eighteen described being able to trust group members completely and felt safe enough to discuss their problems and be vulnerable in front of other group members. *“Within the group setting I trust them all implicitly with my vulnerable child*. *I have no problem allowing my vulnerable child to be there and discuss whatever and allow myself to be vulnerable”* (patient 5002). As discussed in the introduction the vulnerable child here refers to a mode state where the person experiences distress and cognitions associated with negative sense of self such as worthlessness, helplessness, incompetency, mistrust, or abandonment [11]. On the other hand, 10 patients felt unsafe among group members and expressed reluctance to share parts of themselves, particularly after conflict occurred during group. They described initially expressing honest opinions but upon receiving negative responses from particular group members, patients would ‘pull back’ and not contribute as much as before. Patients mentioned that trust had been lost and they felt the need to withhold their opinions for fear of triggering intense emotional responses in others. *“I’ve been at the end of one of these people’s anger outbursts twice… so I’ve learnt to shut up*, *they can lose control quite easily and it’s definitely not safe for me… You can’t talk to these people about it you can’t say*, *‘Well you need to shut up more and let other people talk’ because I mean you’d get your head ripped off*, *you would”* (patient 5230).

#### C3. Feelings that arose when comparing oneself against others

Patient narratives (*n* = 13; 36%) included a mixture of feelings including frustration, irritation and comfort when comparing oneself against others in the group. In particular four patients described feeling frustrated at themselves for not progressing or picking things up as fast as others. *“Especially if someone in the group has a revelation before you do*, *and they come in going*, *‘Guess what*! *I recognized my trigger and I managed to stop it*!*’ You think*, *why can’t I do that*?” (patient 5211). Conversely 10 patients indicated that when group members disclosed their negative experiences/struggles, it provided some sense of comfort/relief, enabling them to feel more at ease to share their own experiences and notice the progress they made. *“I can recognize things that people are describing or the way they act and that would have been me 5 years ago but I’m not there anymore*. *And as awful as that sounds you look at someone who is not doing well and that makes you feel better about yourself*, *that’s also enabled me to see how far I’ve come”* (patient 5206).

#### C4. Gender composition of the group

Male patients were rare in the treatment groups. Eighteen patients (eight males; 10 females) including all 12 participants in the Dutch sample commented on the impact of gender of their group experiences of ST. Apart from two females indicating their discomfort with a male therapist, others had nil issues with the therapists’ gender. In a similar vein, majority of females described the initial unpleasantness being in the presence of males. *“Some things came up of which I thought- I don’t want to hear this- sexual stuff… I feel a bit embarrassed but I thought- accept them how they are*, *basically they are not doing anything wrong… But for me it wasn’t pleasant*. *Women amongst each other is no problem”* (patient 7011). Three male patients (38% of males in this study) found it difficult to engage in open discussions for fear of embarrassment and offending the opposite gender. *“I talk easier with a man about it (*Topics of an intimate/sexual nature) *than with a woman… I sometimes have*, *as a man apologized for what is done to them by other men”* (patient 7439). Having said that, it was also indicated that in the longer term having both genders in the group was helpful, for instance because they learnt that both genders can struggle with similar issues, and because it corrected stereotypical views and distrust.

### D. Structure and format of therapy

The majority of patients (*n* = 27; 75%) commented on the structure and format of ST. Most participants commented on the experience of combining group and individual ST, and roughly half the participants commented on the use of experiential techniques, and most commented on the usefulness of the schema mode model. Most were dissatisfied with the two-year duration of therapy, regarding it as insufficient.

#### D1. Extent to which group and individual ST complemented each other

Twenty-seven patients (75%) discussed the necessity of having a combination of group and individual ST, explaining how each was different yet equally important. Patients found the group component useful in learning and applying schema-related concepts and coping skills in a safe environment where they can practise these skills with others before applying them outside therapy. Seventeen of them also indicated the utility of individual therapy in providing space to ventilate conflicts that occurred during group and discuss sensitive topics or issues on a deeper, more personal level. *“Group focuses on learning the stuff you need in order to get better*, *and one-on-one focuses more on you and why you need this stuff in the first place… In order to utilize the things you learn in group*, *you need to have the one-on-one to work through all that past stuff”* (patient 5211). Moreover, out of all 20 patients in the twice-weekly group format, 14 (70% of the twice-weekly group format) expressed their preference for more individual sessions. They were able to request these individual sessions from the group therapist. None of the patients in the once-weekly group sessions requested more group sessions. The importance of trust was deemed essential in facilitating open conversations in the group setting, and patients found it difficult to open up without additional support from individual sessions. *"With borderliners*, *the disorder mostly has traumas which are deeply embedded*, *and you of course cannot talk about that in the group and the individual is too little for that*. *I am here 1*.*5 years now and I could not talk about it*, *because first you need a long time until trust is built"* (patient 2404). Additional support from individual sessions were more valued at the beginning of therapy and 50% of those who preferred more individual sessions became less bothered by this twice-weekly group ST arrangement as therapy progressed. *“At the start I preferred both group and individual therapy once a week*, *however since dealing with other people*, *I find it really pleasant*. *Of course*, *you receive less personal attention in the group*, *however group members will notice you too*. *It somehow works both ways”* (patient 7205).

#### D2. The use of experiential techniques

Out of 17 patients (47%) who discussed the use of experiential techniques, 15 appreciated the use of imagery rescripting and chair dialogues. The provision of therapy concepts, theories and definitions experientially were perceived as more effective than plainly discussing these as it contributed to a deeper level understanding. Despite some initial reluctance from most patients *“The imagination-exercises were really difficult at the beginning- I was always reluctant… so actually with every imagination-exercise*, *I dissociated away but that also changed at some point- because I then learnt anti-dissociative techniques and stuff”* (patient 2018), the speed and intensity with which patients understood the origins of some of their emotions took them by surprise, leading to an increased capacity to reconceptualize some of their entrenched thought processes. *“The chair work I found really incredible that I could really imagine where these extreme feelings of tension come from- how they crash into you… oh that is scary*! *And these imagination exercises*, *especially for not-so-good memories I think are really great*, *to rearrange them like that*, *that gives you a completely different feeling… all of a sudden they are a lot less hard to bear… and not so negatively loaded anymore”* (patient 2404).

#### D3. Duration/length of therapy sessions

Twenty-two patients (61%) expressed dissatisfaction regarding the length and frequency of ST; half of whom described the two-year duration as insufficient and found the gradual decrease in frequency increasingly difficult because they felt they had not made sufficient progress before therapy formally concluded. *“I’d like to add another year because I have the feeling*, *there are just many steps now*, *some steps I already took*, *but I am not ready yet*. *Over 40 years I lived in certain constraints or in a certain mode and I could break open a little within these 1*.*5 years*, *I am gradually learning to understand myself and to apply the things I learn here but I don’t have the feeling that I can recondition 40 years in these 1*.*5 years*” (patient 2403). A quarter of patients in the twice-weekly group ST format initially found the frequency of twice weekly group ST burdensome. All five patients adjusted well to the arrangement eventually and had difficulty coping with the reduction in group ST frequency. *“At the beginning it was really difficult…it took up a lot of space and I had to arrange that with full-time work*. *And interestingly now*, *when you established it and everything works fine*, *they take that hour from you and so I find it really difficult to deal with the reduction now because they are such pillars”* (patient 2411).

#### D4. The use of schema-mode model

Twenty-one patients (58%) discussed the usefulness of psychoeducation through schema-related content and while two initially described the schema terminology as “confusing” or “difficult to grasp”; they both subsequently found the concepts helpful in categorizing and making sense of various events/situations. *“The structuring of ST- the subdivision*, *how you structure yourself*, *where you are at the moment and why it is like that*. *I find that very helpful*, *to be able to analyze yourself and say ‘Which schemas*, *modes are there*, *and where do they belong*?*’ That we first roughly looked at it then go into detail into every single aspect*. *I found that helpful like a huge poster; what opened up there*. *There were also many aha-moments”* (patient 2454).

#### D5. Email access to therapists outside working hours

Thirteen patients (36%), 11 from the German sample discussed the value of having email contact with therapists outside office hours. Their narratives reflect an increased sense of security and support from this form of therapist accessibility. *“Especially via e-mail- that helped me a lot… I always found that very supportive and also in between the sessions*, *sometimes even in the weekend*, *which really surprised me positively*, *because therapists deserve to have weekend*. *I thought it was huge*, *because the effort was a little bigger there… You know you are not alone in whatever situations*. *You always know you have somebody who maybe looks at it better from the outside than a friend”* (patient 2456).

### E. Therapeutic relationships

Altogether 29 patients (81%) regarded the quality of therapeutic relationships as significant in influencing therapy outcomes. A majority described their individual and group therapists positively while a handful felt otherwise.

#### E1. Extent to which patients feel supported by their therapists

Fifteen out of 20 patients; 75% of those who described their individual therapists used a variety of adjectives including “brilliant”, “clever”, “amazing”, “supportive” and “attuned”. They described a good fit in the therapeutic relationship where they felt emotionally connected and appropriately supported. Two in particular found the corrective experience of the therapeutic relationship essential. *“I somehow say this exaggerated now- but it was a little like a parent-substitution*. *That was extremely important for me because what my therapist reflected or what she mirrored me in that moment; that was what I did not get before… the relationship between therapist and patient in my opinion is the most important thing for the whole ST to work”* (patient 2018). On the other hand, the other five patients expressed feelings of dissatisfaction and frustration, particularly because they felt misunderstood. *“She was making an assumption on what she thought was wrong and what needed to be fixed- but that’s not necessarily what I think is wrong or needs to be fixed”* (patient 5206). Seventeen patients found group therapists supportive and helpful, both during and outside of group therapy sessions and described them as non-imposing and non-judgmental in their views, which allowed them to freely speak their minds. Group therapists were described as attuned to whatever was happening within the group creating a sense of safety. *“They’re onto you*. *Even if I well up with tears and I don’t actually drop one*, *I’m already noticed*. *If I shift in a chair*, *they know I’m in pain*. *One of them could be talking*, *but the other one’s keeping an eye on you*. *That brings a safe feeling in”* (patient 5025).

## Discussion

### Main findings

The purpose of this study was to understand the experience of ST for individuals with BPD. Reported gains following the two-year-long ST program included increased insight, better connection with one’s emotions, improved self-confidence, increased cognitive flexibility in terms of taking alternative perspectives and being less harsh to oneself. These reported benefits lend support to previous quantitative studies where ST was found to improve therapy outcomes such as better quality of life, reduced BPD symptomatology and positive changes in maladaptive schemas [16, 17, 19]. While patients’ definitions of recovery have not been directly explored in this study, aforementioned improvements made were consistent with various representations of recovery from BPD found in a qualitative study based on 48 service users [30] and in a recent meta-analysis exploring the areas of improvement regarded as important in achieving recovery [23]. In these studies, recovery from BPD was characterized by enhanced confidence, better self-understanding, reduced self-blame and greater self-acceptance, consistent with findings in the present study. A range of psychological interventions including cognitive behavioural therapies, psychodynamic approaches, integrative approaches, standard mental health services, and specialist services such as DBT and MBT were offered; treatment characteristics identified as helpful include setting boundaries and focusing on change such as problem-solving [23, 30]. In the current study, patients’ narratives of ST affording greater focus on internal processes; i.e. making sense of the origins behind one’s thoughts and feelings, with more consideration of the uniqueness of one’s background indicate the differential areas of focus between ST and other treatments (e.g. retrieving and healing aspects of one’s past experiences). Characteristics specific to the ST model have been identified in facilitating the aforementioned improvements.

#### Therapeutic gains attributed to components of the schema model

Components within the schema model identified as facilitating positive outcomes included: the use of experiential techniques, schema-related concepts (such as schema modes), therapeutic relationships and the complementary nature of group and individual ST. The finding that experiential exercises facilitate one’s capacity to take alternative perspectives suggest that information is more effectively processed in the presence of affect and emotional experiences (pleasant or unpleasant). Patient responses to imagery rescripting in the present study were largely positive, including a sense of surprise where the experience exceeded expectations. On the other hand, in a study that explored patients’ perspectives of imagery used in the initial phases of ST, patients with cluster C personality traits indicated unpleasant feelings of anxiety, fear and annoyance [31]. The disparity could be due to the duration of time patients underwent ST before being interviewed and therefore the differing nature of imagery used; first three months into therapy with the use of diagnostic imagery and safe place imagery [31] versus over 12 months of therapy with the additional use of imagery rescripting in the present study. It is noted patients in the present study tended to recognize the benefits of these exercises in retrospect and patients in the imagery study [31] also reported better understanding the use of imagery as they progressed further in therapy.

The usefulness of schema-related concepts in explaining the origins of current behaviour patterns and clarifying the mechanisms behind one’s reactions are consistent with the aims of the education phase of ST [8]. In contrast to psychoeducational interventions which focus on providing information about symptoms and how to manage them [32], the education phase of ST emphasizes on how modes or schemas came about during the person’s development and subsequently influence life patterns. The distinction while subtle is less stigmatizing because it encompasses one’s personality structure rather than limiting the focus on the disorder. Most patients in the study found ST more effective than previous therapies albeit difficult primarily due to the depth and level of understanding with which ST works through as well as its consideration of one’s past experiences and uniqueness of individual needs. These themes signify a therapy experience that extends beyond the circumscribed focus on borderline symptoms to consider factors that contribute to one’s personality structure and sense of self. Symptoms that characterize the borderline diagnosis have often been referred to as a consequence of the disorder rather than an attempt to maintain some sense of personal integrity in response to trauma [33, 34]. An overemphasis on treating borderline symptoms can inadvertently overlook the existence and extent of past abuse and over-pathologize adaptive reactions to valid psychosocial stressors. In ST, symptoms and other problems are framed as attempts to deal with unmet childhood needs. This approach promotes self-understanding and a sense of continuity from childhood [8], similar to previous findings of ST with cluster C personality traits [35].

Patients’ experiences with their group and/or individual schema therapists in the study identified non-judgemental attitudes and attentiveness to their needs as therapist qualities that enable a sense of safety and a strong therapeutic relationship. While there is no guideline specifying the type of therapist characteristics needed for ST, the therapist role in ST involves limited reparenting, which relates to providing emotionally corrective experiences for patients by the therapist meeting their core needs that were missed in childhood within professional boundaries. Flexibility in adapting to meet each patient’s emotional needs is recommended so that the patient can develop healthy behaviours modelled after the therapist [8]. A high quality therapeutic alliance forged during the early phases of ST up to three months of treatment was found to be predictive of lower treatment termination rates and significant reduction in the degree of patients’ maladaptive beliefs at later phases [35, 36]. It is of benefit to further examine patients’ experiences of and/or what they value in the therapy alliance as they help clarify what patients regard as important in a schema therapist.

Three quarters of the study participants commented on the benefits of having both group and individual schema therapy. This finding is consistent with earlier studies that found schema therapy to be more effective when delivered in tandem [20, 21]. The considerable preference for more individual sessions in the twice-weekly group ST format in this study further highlights the value of individual therapy as a supplement to group sessions in supporting patients to open up. Findings specific to the twice-weekly group ST format also illuminate patients’ initial discomfort with the intensity of twice weekly group sessions. Even though majority eventually overcame this, increased one-on-one contact particularly at the beginning of therapy potentially serves to increase the ease and pace with which patients adjust to the arrangement. While recovery from BPD is possible with group ST alone, present findings indicated synergetic effects where individual sessions can help the patients benefit from the group; by addressing concerns about the group and allowing for more complex experiential work and deeper exploration of their schemas.

#### Group factors that contribute to therapeutic gains

Patients’ reported experiences in the group were consistent with some therapeutic group factors including universality and instillation of hope [37]. In contrast to the expectation that witnessing group members who improved as a result of therapy could instil hope in others [37], present findings revealed some patients’ self-directed frustration for not progressing as quickly as their peers, yet feeling better about themselves when comparing to those who self-disclosed life struggles. This finding may reflect the Failure schema common in BPD patients, where one feels inadequate relative to their peers [8]. The contrast in findings could be due to the type of therapy and/or differences in the sample characteristics (cancer patients [37]; BPD patients in this study). BPD patients tended to be punitive towards themselves [8, 38] and therefore engage more easily in self-criticism.

#### Factors that disrupt group climate

The finding that a particular incident/conflict involving a few patients left others feeling unsafe to speak their minds and subsequently contributing less suggests that such events can stifle personal growth. It also implies that tension could linger even if a conflict appeared to be resolved. It has been proposed that conflict is essential to group development because ability to effectively deal with conflict contributes to individual maturation [39, 40]. However it becomes a problem when the conflict is not adequately managed to the extent that it compromises group members’ sense of safety [41]. In the treatment protocol, there is an emphasis on maintaining safety early on in the group’s life and this includes limiting and/or managing conflict effectively [42]. The finding that group safety was compromised following conflict highlights the challenges in managing group conflict successfully. This is particularly so in group therapy with BPD patients [42], indicating the need for special attention in therapy training and supervision in this respect.

#### Termination of schema therapy

Unsurprisingly as the ST program approached its end, some patients found the tapering of group and individual sessions disruptive and had difficulties with each transition of session frequency from weekly to fortnightly and then monthly. Regardless of the therapy approach, termination can be a challenging process filled with a sense of loss and pain, particularly for the BPD patient who experiences a greater sensitivity to such issues [43]. For these reasons negotiating an end date with BPD patients, addressing termination concerns well before its end, building up their resources and ensuring therapists’ availability in times of crisis and important events in patients’ lives have been suggested [14]. However, negotiating personalized tapering of sessions seems more difficult within a group format. In the current treatment protocol, all groups had a pre-set end date and the early discussion of termination was emphasized. Patients were also encouraged to identify what supports them to feel less abandoned and the groups focused on accomplishments and benefits with each reduction in the frequency of sessions.

### Differences among countries

There existed some variances across sites, with some themes more pronounced in a specific country. For example, a significantly large proportion of the finding on group gender composition was from the Dutch cohort, where all Dutch participants commented on the gender issue. This may be attributed to the presence of male group schema therapists and higher number of male patients in the Dutch groups; five male patients compared to those at the Australian and German sites; two and one male patients respectively. Given the finding that every patient in the Dutch cohort commented on gender, five of them were approached and interviewed further on this topic after coding of the current sample was completed. This data was analyzed as part of a master’s thesis [44], and findings from these interviews did not lead to any additional insights to those reported here.

Additionally, the finding that patients value therapist accessibility via email outside office hours was particularly endorsed in the German interviews as compared to the Australian and Dutch ones. This could be due to the implementation of after-hours therapist contact within the ST protocol in Germany. While current findings reflect a positive patient experience of additional therapist contact, a previous study found no significant increase in BPD symptom reduction by adding additional therapist telephone availability to individual ST [18]. This discrepancy may be attributed to the different means of therapist access; email contact in this study versus telephone contact [18]. Even though study patients were given the choice of communication via phone and email, those who utilized this option indicated their preference for email contact. Constructing an email involves an aspect of writing and cognitive processing. Past research has documented the psychological benefits of writing about stressful events, where such positive effects may be related to developing a cognitive narrative and facilitating organisation and structure to an otherwise chaotic internal world [45, 46].

### Strengths and limitations

There are several strengths regarding the methodology and data analysis. First, the sample size of 36 from multiple sites in different countries ensured that data saturation was achieved and themes emerged were not restricted to idiosyncrasies of patients at any individual site or country. Second, the research team was involved in various stages of the data analysis process where multiple coding was carried out allowing for thorough double coding and crosschecking of data. Third, the informant feedback used could correct any misinterpretation of the interview data, minimizing interviewer subjectivity. Finally the coding frame had a high degree on inter-rater reliability and the interviewers were not trained schema therapists, reducing any biases.

On the other hand, the length of time patients had undergone ST by the time of interview was not controlled for. There may be latent differential experiences consistent with the respective formats and time points during the course of ST. Additionally even though all the German and Dutch interviews were translated to English, a certain degree of meaning may have been lost in translation.

### Clinical implications and directions for future research

This inquiry is an initial attempt to understand holistically how various components embedded within the schema framework are experienced by BPD patients. These findings while exploratory in nature contribute to current understandings and address limitations of the schema model while enhancing its feasibility and implementation. Treatment characteristics identified that could better inform future delivery of ST include but are not limited to:

1. The delivery of ST with a combination of group and individual formats was described as beneficial for BPD patients in this study. Perhaps individual ST alone may be limited in providing a realistic environment to apply and experience new ways of interacting with others while group ST alone may not afford sufficient space for patients to explore their issues at a deeper, more personal level.
2. It is of benefit to re-evaluate the twice-weekly group ST format with consideration of facilitating better access to and increasing the number of available individual sessions, particularly at the beginning of therapy.
3. Patients’ experienced anxiety and frustration about not recovering fast enough suggest the value of pre-emptively exploring their expectations and beliefs early in the group, as it relates to schemas such as defectiveness and failure.
4. Paying close attention to group process is essential particularly to silent factors (e.g. disengagement) and presence of aggressive behaviours which appears to trigger withdrawal from others. This is highlighted in the training and delivery of GST, nonetheless patients still reported fear of being at the receiving end of intense emotional responses. Results of this study therefore suggest that aggression and threatening venting of personal anger by one group member to another should be limited by therapists as soon as possible, for which they need to act firmly and in an appropriate way. Waiting with limit setting might leave a memory trace that is difficult to counter in later sessions. It might also be that GST is contraindicated for a subgroup of BPD patients; not because they suffer from anger problems, but because they have such difficulties in controlling aggression and angry verbalizations and accepting therapists’ attempts to limit these. It is possible such patients are too much of a threat for the safety of the group as a whole. The challenge is to find a way to validly detect these patients before treatment commences. The present study excluded Antisocial Personality Disorder (ASPD) and Narcissistic Personality Disorder (NPD), but there are indications that this was not enough of a safeguard against such problematic behaviours. This issue of angry behaviour interfering with treatment outcome for BPD is not unique to ST. Indeed a structured interview for anger and assertion has been found to mediate treatment response for BPD patients receiving DBT in a group setting [47]. Perhaps individuals high in hostility are better managed in an individual ST format.
5. It is important to begin the discourse early regarding termination to facilitate an open discussion of patients’ concerns. Personalized tapering of individual sessions might also be considered.

The research has attempted to capture BPD patients’ experiences of ST. The course of receiving ST has evidently contributed to meaningful progress in their lives. Identified specific mechanisms of the schema model and non-specific therapy ingredients responsible for these gains will not only create opportunities for improving the existing ST protocol but also enhance current understandings of their interactions that help shape recovery from BPD. Future research could consider exploring the interplay between effects of additional training for group schema therapists and how level of patient dysfunction within the group has an impact on group therapists’ ability to manage conflict. Health professionals working within the schema model could also develop customized treatment plans taking into consideration individual differences and personal treatment goals.
